# Sinking Skin Flap Syndrome After Decompressive Hemicraniectomy in a Patient With Calvarial Multiple Myeloma Who Underwent a Lumbar Puncture: A Case Report

**DOI:** 10.7759/cureus.24458

**Published:** 2022-04-25

**Authors:** Sara Tonini, David Jordanovski, Karlene Williams

**Affiliations:** 1 Internal Medicine, Overlook Medical Center, Summit, USA

**Keywords:** neuro radiology, lumbar puncture (lp), craniectomy, multiple myeloma and cns involvement, sinking skin flap syndrome

## Abstract

Sinking skin flap syndrome (SSFS) or “syndrome of the trephined” is a rare complication that can occur after decompressive craniectomy. Disabling neurologic deficits, as well as the impairment of overall mental status with the development of a concave deformity and relaxation of the skin flap, are frequently observed. This usually develops several weeks to months after craniectomy. The pathophysiology of the syndrome includes cerebrospinal fluid (CSF) hypovolemia and the development of an atmospheric pressure gradient that can be worsened by CSF diversion, dehydration, and change in position such as can be seen with a lumbar puncture. We present a case of a 40-four-year-old male with calvarial multiple myeloma three months after craniectomy who developed SSFS two days after lumbar puncture was performed to investigate possible leptomeningeal spread. It is imperative to recognize the syndrome early and proceed with urgent management with measures that initially increase intracranial pressure such as IV hydration and Trendelenburg positioning. In certain cases, proceeding with surgical management, such as epidural patch or cranioplasty, can be life-saving.

## Introduction

Bone defects of the skull are seen in many different pathological conditions. These can include head trauma and conditions, such as a tumor mass or cerebral edema, which require surgery of the skull. The bone defect itself can be the cause of SSFS independent of the cause that required the craniectomy. Despite the importance of early recognition of neurological symptoms related to this syndrome, the description of SSFS has relied on a limited number of case reports [1].

Reported cases of SSFS do not seem to show any association of this syndrome with a lumbar puncture. Zhao et al., in only one case report, mention SSFS following decompressive hemicraniectomy and lumbar drainage. Lumbar drainage was performed in a patient with subarachnoid hemorrhage.

We present a case of a patient who developed SSFS having undergone decompressive hemicraniectomy for multiple myeloma with skull and epidural tumor excision three months prior and two days after receiving lumbar puncture for the evaluation of leptomeningeal spread. We postulate that the development of this syndrome may be related to lumbar puncture, a delayed complication of craniectomy, or a combination of both.

## Case presentation

**Figure 1:**
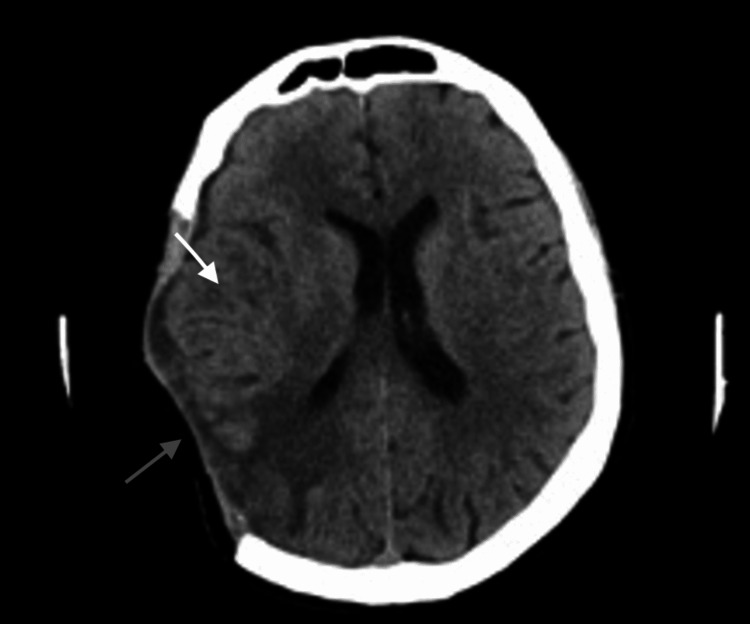
Computed tomography angiogram scan of the head showing postoperative changes (gray arrow) with right hemispheric edema similar to prior studies (white arrow)

A 44-year-old Hispanic male with a history of multiple myeloma of the right parietal calvarium (extra-axial) treated with decompressive hemicraniectomy and partial epidural tumor resection three months before admission presented to the hospital due to worsening left-sided weakness, worsening headaches, ptosis, blurry vision, and diplopia. The patient had recently been discharged after completing a full round of inpatient chemotherapy with cyclophosphamide, bortezomib, and dexamethasone and was to be transitioned outpatient to daratumumab - lenalidomide/bortezomib and dexamethasone. Vital signs upon presentation were within normal limits other than an elevated pulse of 103 bpm. Physical examination was significant for new-onset left lateral rectus palsy and weakness of superior rectus and inferior oblique muscles, a left upper extremity weakness with deltoid 2-3/5, biceps 3-4/5, triceps 4/5, handgrip 3-4/5, decreased hand intrinsics, left lower extremity with quadriceps 4/5 iliopsoas, dorsiflexion, plantarflexion, extensor hallucis longus 4-5/5, right upper extremity, as well as right lower extremity strength was 5/5. The sensory exam was intact. Glasgow Coma Scale (GCS) was 15. Labs, including a complete blood count and a complete metabolic panel, were within normal limits. CT head (CTH) was done, which showed postoperative changes with right hemispheric edema in a similar pattern compared to previous studies (Figure 1).

**Figure 2:**
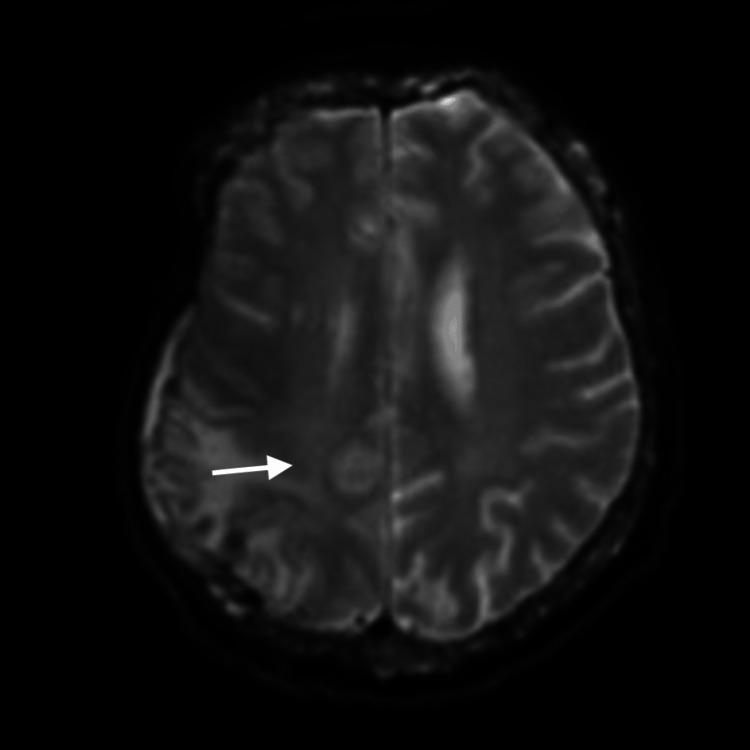
Magnetic resonance scan of the brain showing a small region of diffusion signal within the right parietal lobe concerning acute infarct (white arrow) An interval decrease in the size of previously visualized right parietal mass with a decrease in right parietal lobe vasogenic edema and a decrease in the right to left midline shift to 2.5 mm is seen.

MRI brain without contrast showed an interval decrease in size of the previous right parietal mass with an overall decrease in vasogenic edema within the right parietal lobe and had a decrease in the right to left midline shift to 2.5 mm (Figure 2).

**Figure 3:**
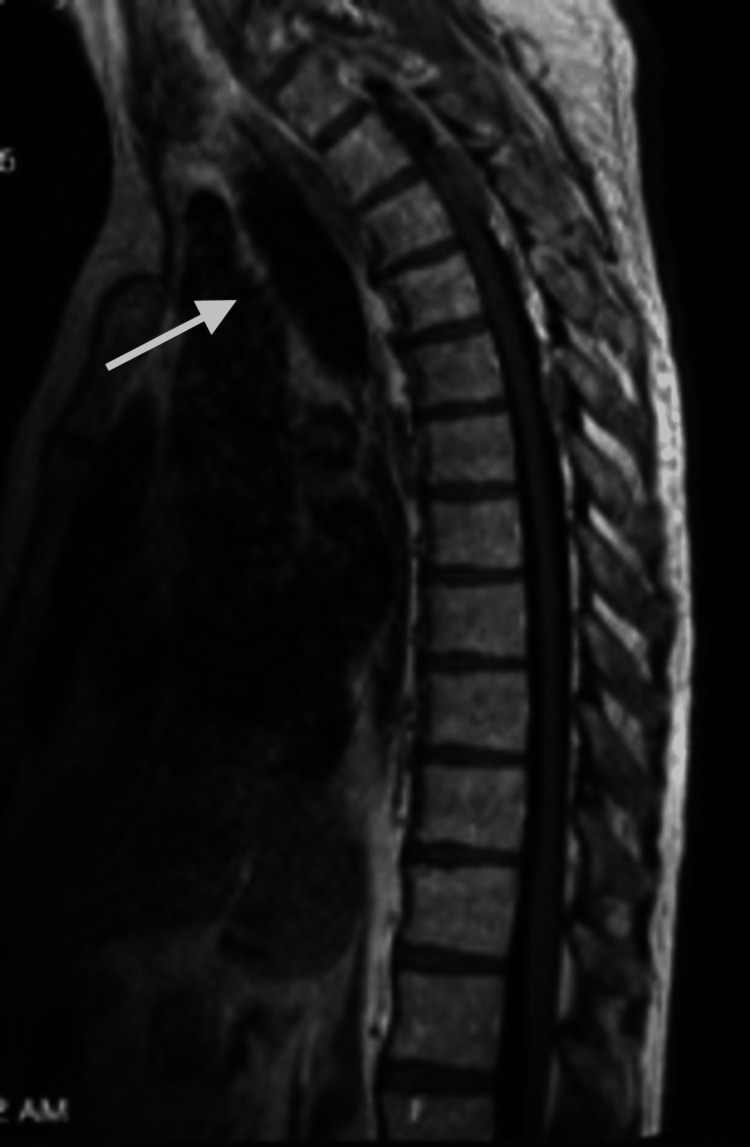
MRI thoracic spine w/wo contrast showing an epidural hematoma extending along the posterior aspect of the thoracic spine from T2-T6, left greater than right and most prominently at the level of T3 where there is mild mass effect over the left posterior spinal cord (white arrow)

MRI cervical, thoracic, and lumbar spine showed a mass in the T3 area measuring 2.6 cm x 1.6 cm x 1.1 cm extending from T2 to T6 causing moderate spinal canal stenosis with compression concerning for leptomeningeal spread (Figure 3).

No frank spinal cord compression was seen on imagining. A lumbar puncture was planned to evaluate for possible leptomeningeal spread. He was started on intravenous dexamethasone. Palliative radiotherapy was initiated to control tumor-related pain in the T2-T6 and T10-T12 regions. Lumbar puncture was performed at the L2-3 level with an opening pressure of 8 cm H20 and 12 cc of clear cerebrospinal fluid (CSF) was collected. The CSF analysis is shown in Table 1.

**Table 1:** CSF analysis and normal reference ranges

CSF Analysis	Result	Reference Range
Protein mg/dL	150	12-60
Glucose mg/dL	74	40-70
Neutrophils %	14	0-6
Lymphocytes %	76	40-80
RBC /uL	1000	0
WBC /uL	6	0-5
Color	Pink	Colorless
Xanthochromia	Absent	Absent
Opening pressure cm H2O	8	7-18
Flow cytometry	No plasma cell dyscrasia	N/A

**Figure 4:**
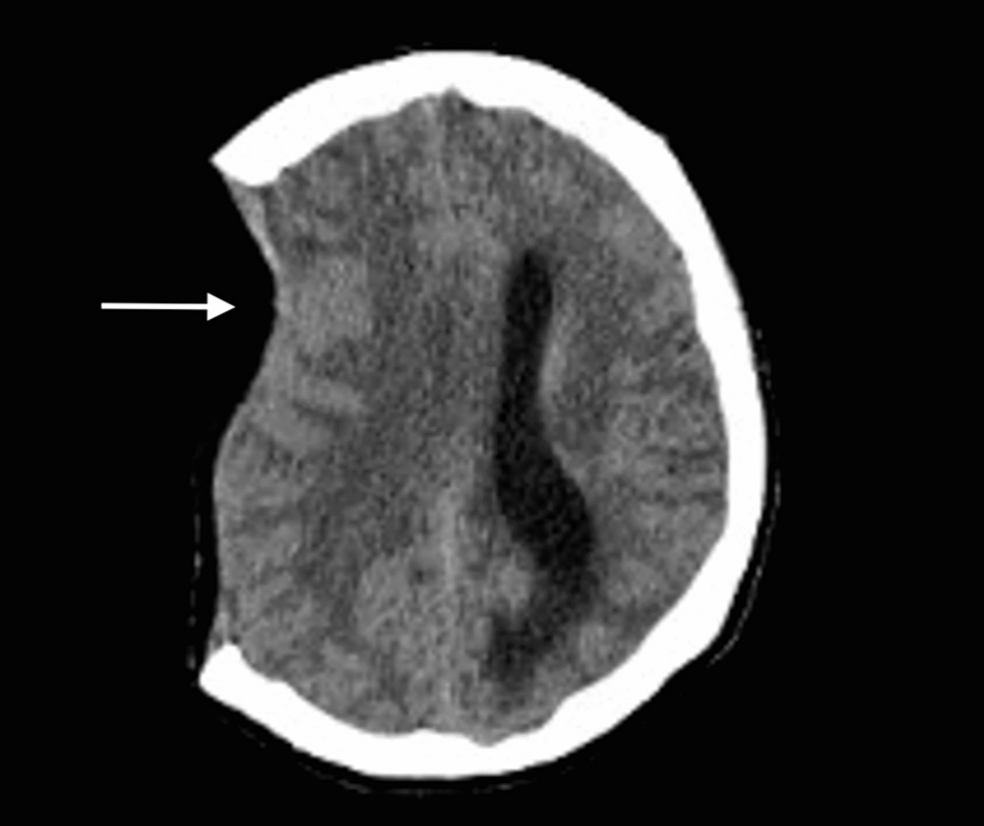
Computed tomography scan interval development of sunken flap syndrome with 11 mm of a leftward midline shift, crowding of the perimesencephalic cisterns, and left lateral ventricular trapping (white arrow)

**Figure 5:**
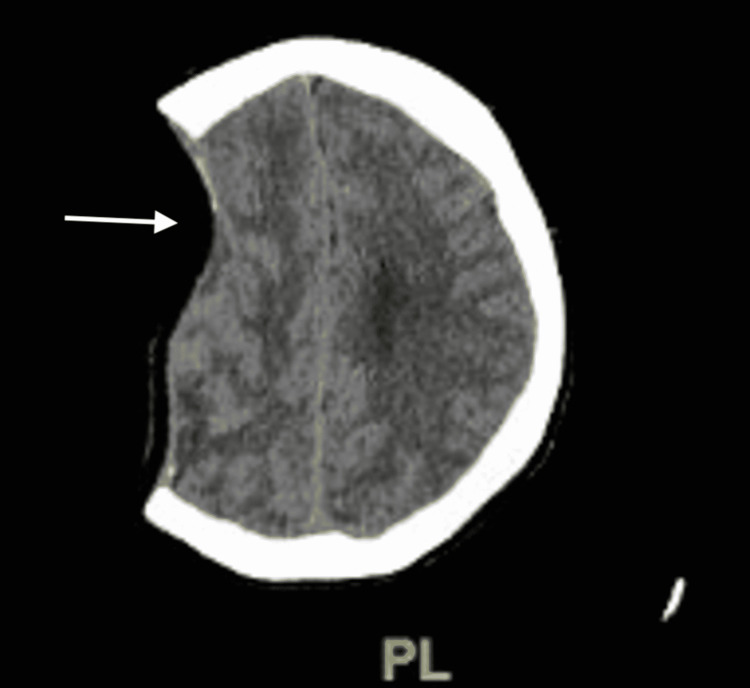
Computed tomography scan Interval development of sunken flap syndrome with 11 mm of leftward midline shift, crowding of the perimesencephalic cisterns, and left lateral ventricular trapping (white arrow)

Flow cytometry was also performed, which showed no overt immunophenotypic evidence of a plasma cell dyscrasia. Two days after the procedure patient became progressively more lethargic, arousable to sternal rub, and oriented, with Glasgow Coma Scale 12, other neurologic exams were unchanged. Repeat CTH was done and showed an increased midline shift of 11 mm with compression of the left frontal and mesencephalic cisterns consistent with SSFS (Figures 4-5).

Surgical intervention was not pursued due to an extremely poor prognosis. Considering his multiple myeloma, progression of his disease, poor response to chemotherapy, as well as worsening neurological status, he was transitioned to hospice care and expired two days later.

## Discussion

Among the complications of craniectomy, SSFS is a delayed complication that occurs when atmospheric pressure exceeds intracranial pressure.

This syndrome is a rare complication that usually occurs after a large craniectomy. The reason for its occurrence is the conversion of the cranium from a closed to an open box during the procedure. Differences between atmospheric and intracranial pressures can occur. When the atmospheric pressure exceeds the intracranial pressure and the skin flap presses on brain tissue, this can cause a paradoxical herniation. Symptoms may include headaches, mental status changes, seizures, focal deficits, and dysautonomia [2].

The pressure-volume relationship, known as the Monroe-Kellie doctrine, affirms that the cranial compartment is incompressible, and therefore the volume that is inside the skull is fixed. Thus, all components of the cranium such as blood, brain tissue, and CSF are in a state of equilibrium with one another such that any increase in one component must be matched with the same intensity decrease in another. In SSFS, neurologic changes are associated with alteration of the pressure/volume relationship in which these components lose their equilibrium, thus resulting in a significant pressure change and the resulting sinking of the skin flap seen on imaging [3].

In the Sarov et al. DEcompressive Craniectomy In MALignant MCA Infarction (DECIMAL) trial, SSFS either clinically symptomatic or asymptomatic affected one-fourth of patients three to five months after hemicraniectomy was performed for malignant middle cerebral artery infarction. Early diagnosis is therefore key to avoiding progression to paradoxical herniation. Associations were found with SSFS in patients with a smaller surface of craniectomy, an older age, a longer delay to receiving cranioplasty, and a tendency to a larger area of infarct [4].

This syndrome is characterized by neurological changes associated with alteration of the pressure/volume relationship between intracranial pressure and the volume of CSF, blood, and brain tissue in patients with large bone defects [3]. Major clinical features include the sunken appearance of skin over skull defect, orthostatic headache, focal neurologic deficits, seizures, and altered mental status. Without any intervention, paradoxical brain herniation, coma, and death may occur [4-5].

Without the rigid structure of the cranium, patients are at risk of developing a pressure gradient across the soft tissues. We postulate that with a lumbar puncture, there is an alteration of the CSF hydrostatic column, which can cause brain tissue to shift as atmospheric pressure exceeds intracranial pressure, which is the main cause of SSFS. 

In our case, for three months post-craniectomy, the patient had no complications but two days after the lumbar puncture, the patient had a sudden alteration in mental status and the CT head showed an 11-mm leftward midline shift, crowding of perimesencephalic cisterns, and left lateral ventricular trapping, which was consistent with SSFS. We speculate the cause could have been both provoked by the lumbar puncture, spontaneous as part of the delayed complication of hemicraniectomy, or a combination of both.

As the forces responsible for generating paradoxical herniation/skin flap syndrome arise from a low-pressure system, the focus of management is to increase the intracranial pressure. Initial measures such as positioning the patient with the head of the bed down, hydration, and discontinuation of both diuretics and hypertonic solutions are considered the standard of care. Other measures include epidural blood patch and cranioplasty.

Symptoms such as focal neurologic deficits, headaches, and altered mental status are regressive after recumbency or epidural blood patch and can resolve completely after cranioplasty. Unfortunately, our patient was not a candidate for an emergent neurosurgical intervention. The initial treatment for SSFS, however, should include supportive measures and shared management with neurosurgery. Trendelenburg positioning is recommended. Urgent operative intervention is rarely indicated; however, in patients who do not improve with conservative treatment, early cranioplasty is most effective in relieving symptoms [6]. Early cranioplasty remains a life-saving measure to perform when midline shifting is confirmed by CT and does result in better outcomes for these patients [7].

Table 2 lists the details of the literature review, which revealed several case reports in which drainage of CSF preceded the development of SSFS.

**Table 2:** A literature review reveals several case reports in which drainage of CSF preceded the development of SSFS CSF: cerebrospinal fluid; SSFS: sinking skin flap syndrome

Study, Authors, Year	Procedure undergone	Time of onset	Outcome
Sinking skin flap syndrome and paradoxical herniation secondary to lumbar drainage. Zhao et al. 2015 [8]	Decompressive hemicraniectomy and lumbar drainage	Several weeks to months	11% mortality rate (1/9 patients died from the paradoxical herniation, 8/9 recovered with conservative management)
Sinking skin flap syndrome: Phenomenon of neurological deterioration after decompressive craniectomy. Khan et al. 2018 [2]	Large left-sided craniectomy with bone flap placement and VP shunt	1 year	Cranioplasty advised – family deferred, VP shunt adjusted to increase intracranial pressure, repeat CT stable midline shift with no interval changes, mental status improvement
Sinking skin flap syndrome with a delayed dysautonomic syndrome – An atypical presentation. Romero et al. 2013 [9]	Decompressive craniectomy for hematoma evacuation and VP shunt	15 months	VP shunt revision and change in medium pressure valve, conservative management, improved neurological condition six months later
Sinking skin flap syndrome after craniectomy in a patient who previously underwent ventriculoperitoneal shunt. Kim et al. 2012 [10]	Craniectomy VP shunt	4 months	Shunt catheter tie – patient recovered health to baseline
Sinking skin flap syndrome in glioblastoma. Matsuoka et al. 2014 [11]	Two craniotomies, placement of VP shunt	2 months and 1 week	Cranioplasty and revision of left VP shunt – gradually improved over the first 72 hrs

## Conclusions

We presented a case of SSFS with delayed presentation three months after hemicraniectomy and two days after lumbar puncture. Physicians should be aware of this complication, as well as of the possible available management options, no matter the period since cranial surgery.

The management of SSFS and paradoxical herniation requires measures that increase intracranial pressure such as Trendelenburg position, IV hydration, clamping of CSF drainage, and discontinuation of hyperosmolar measures. In the absence of paradoxical herniation, SSFS may respond to IV fluid administration and supine positioning, with the head turned down to the side of craniectomy. Cranioplasty, however, remains the definitive treatment when the syndrome is confirmed on imaging. The first steps are always supportive and should not be underestimated, as they can be life-saving.
